# Innate and Adaptive Immune Defects in Chronic Pulmonary Aspergillosis

**DOI:** 10.3390/jof3020026

**Published:** 2017-05-29

**Authors:** Felix Bongomin, Chris Harris, Philip Foden, Chris Kosmidis, David W. Denning

**Affiliations:** 1 Faculty of Biology, Medicine and Health, The University of Manchester, Oxford Rd, Manchester M13 9PL, UK; felix.bongomin@manchester.ac.uk; 2 The National Aspergillosis Centre, 2nd Floor Education and Research Centre, University Hospital of South Manchester, Southmoor Road, Manchester M23 9LT, UK; chris.harris@uhsm.nhs.uk (C.H.); Chris.Kosmidis@uhsm.nhs.uk (C.K.); 3 Department of Medical Statistics, University Hospital of South Manchester, Manchester M23 9LT, UK; philip.foden@manchester.ac.uk; 4 Manchester Academic Health Science Centre, 2nd Floor Education and Research Centre, University Hospital of South Manchester, Southmoor Road, Manchester M23 9LT, UK

**Keywords:** chronic pulmonary aspergillosis, innate and adaptive immune defects, underlying lung disease

## Abstract

We evaluated the expression of biomarkers of innate and adaptive immune response in correlation with underlying conditions in 144 patients with chronic pulmonary aspergillosis (CPA). Patients with complete medical and radiological records, white cell counts, and a complete panel of CD3, CD4, CD8, CD19, and CD56 lymphocyte subsets were included. Eighty-four (58%) patients had lymphopenia. Six (4%) patients had lymphopenia in all five CD variables. There were 62 (43%) patients with low CD56 and 62 (43%) patients with low CD19. Ten (7%) patients had isolated CD19 lymphopenia, 18 (13%) had isolated CD56 lymphopenia, and 15 (10%) had combined CD19 and CD56 lymphopenia only. Forty-eight (33%) patients had low CD3 and 46 (32%) had low CD8 counts. Twenty-five (17%) patients had low CD4, 15 (10%) of whom had absolute CD4 counts <200/μL. Multivariable logistic regression showed associations between: low CD19 and pulmonary sarcoidosis (Odds Ratio (OR), 5.53; 95% Confidence Interval (CI), 1.43–21.33; *p* = 0.013), and emphysema (OR, 4.58; 95% CI; 1.36–15.38; *p* = 0.014), low CD56 and no bronchiectasis (OR, 0.27; 95% CI, 0.10–0.77; *p* = 0.014), low CD3 and both multicavitary CPA disease (OR, 2.95; 95% CI, 1.30–6.72; *p* = 0.010) and pulmonary sarcoidosis (OR, 4.94; 95% CI, 1.39–17.57; *p* = 0.014). Several subtle immune defects are found in CPA.

## 1. Introduction

The saprophytic, opportunistic, and ubiquitous airborne moulds of the genus *Aspergillus* are capable of causing a wide spectrum of bronchopulmonary diseases in a range of susceptible individuals [1]. *Aspergillus fumigatus* is the usual culprit; *A. flavus*, *A. niger*, and *A. terreus* are the other three clinically significant species encountered in mycology settings of the over 300 authenticated species of *Aspergillus* [2,3]. Invasive pulmonary aspergillosis (IPA), characterised by hyphal angioinvasion, is a life-threatening infection that occurs in severely immunocompromised patients with prolonged and/or profound neutropenia or T-cell dysfunction [4]. In the hypersensitive host, conidia inhalation initiates an allergic response culminating in allergic bronchopulmonary aspergillosis (ABPA) in individuals with asthma or cystic fibrosis [5]. Saprophytic colonisation occurs in immunocompetent individuals with anatomical lesions in their airways or pre-existing architectural lung disease usually following healed pulmonary tuberculosis [6]. Semi-invasive pulmonary aspergillosis (SIPA), previously termed chronic necrotizing pulmonary aspergillosis (CNPA), is a gradually progressive disease (<3 months) described in patients with mild systemic immunodeficiency such as human immunodeficiency virus (HIV) infection, diabetes mellitus, and patients on chemotherapy or immunosuppressive therapy, as well as chronic lung pathology in many patients [7,8].

Chronic pulmonary aspergillosis (CPA) refers to a spectrum of syndromes from simple aspergilloma to slowly progressive destruction of the lung parenchyma with expansion of the existing cavity or creation of new cavities, a condition termed chronic cavitary pulmonary aspergillosis (CCPA). CCPA can progress to extensive pleural thickening or fibrosis of the lung tissue with loss of lung function, an end-stage disease condition termed chronic fibrosing pulmonary aspergillosis (CFPA) associated with significant morbidity and mortality [9]. Previously treated pulmonary tuberculosis (TB) or non-tuberculous mycobacterial (NTM) infections are the most frequent predisposing factors to CPA. The other underlying diseases complicated by CPA include: bronchiectasis, chronic obstructive pulmonary disease (COPD), ABPA, fibrocystic pulmonary sarcoidosis, hyperimmunoglobulin E syndrome, rheumatoid arthritis, and ankylosing spondylitis [10,11]. With all these respiratory co-morbidities, it is not surprising that the estimated global burden of CPA is nearly three million worldwide [9,12].

In the normal host, synergistic and highly co-ordinated interaction between the innate and adaptive arms of the immune system ensures that inhaled *Aspergillus* conidia do not cause disease [13]. Alveolar macrophages, neutrophils, and peripheral blood monocytes are the first line of phagocytic host anti-*Aspergillus* defences to inhaled conidia and hyphae [4,14]. Cytokine-mediated recruitment of natural killer effector cells is necessary for effective host defence against *Aspergillus* infection [15]. Additionally, a number of membrane-bound and soluble pathogen-recognition receptors (PRRs) that identify fungal motifs including toll-like receptors (TLRs), dectin-1, surfactant proteins A and D, mannose-binding lectin (MBL), and pentraxin-3 are important triggers of innate immune response leading to complement activation, phagocytosis, and killing of ingested fungi [16,17]. Activation of PRRs induces maturation of dendritic cells that prime and direct adaptive immune response. CD4^+^ T-cell differentiates into type 1, 2, and 17 helper T-cells, T_H_1, T_H_2, and T_H_17, respectively [18]. T_H_2 cells produce interleukin (IL)-4, IL-5, and IL-13 and are implicated in the pathobiology of ABPA [5,19]. *Aspergillus*-specific T_H_1 and T_H_17 cells produce cytokines such as interferon gamma (IFN-γ) and IL-17 which facilitate macrophage activation and neutrophil recruitment, respectively [16].

Both inherited (genetic) and acquired aberrations in these complex networks of interactions in antifungal immunity, coupled with local pulmonary epithelial compromise predispose to the development of a variety of pulmonary syndromes in response to *Aspergillus* [8,16,20]. Defects in surfactant proteins, impaired production of IL-10, IL-12, IL-17, and IFN-γ together with MBL, TLR, and transforming growth factor-β_1_ (TGF-β_1_) polymorphisms in the host genes that mediate innate immunity have been shown to increase susceptibility to *Aspergillus* infection [20,21,22,23,24,25].

Although immune defects have been well characterised in the IA population, they have not been assessed as contributing to the pathogenesis of CPA. This study evaluates the level of expressions of biomarkers of T-cells (cluster of differentiation (CD) 3, CD4, and CD8), B-cells (CD19) and natural killer cells (CD56), immune homeostasis (CD4:CD8 ratio), and associated factors in a large population of patients with CPA.

## 2. Materials and Methods

### 2.1. Study Locale and Design

This was a retrospective study (audit) involving analysis of secondary data obtained from 144 CPA patients attending the National Aspergillosis Centre (NAC), University Hospital of South Manchester (UHSM), United Kingdom (UK). NAC is a large highly specialised tertiary centre dedicated to the clinical care of CPA patients and others with aspergillosis referred from all over the UK. Patients referred from 1 April 2009 to July 2016 were included in this audit.

### 2.2. Patient Selection

We collected all the available results of immunological biomarkers of T, B, and natural killer cells, a panel consisting of cluster of differentiation CD3, CD4, CD8, CD19, and CD56. This is a standard panel of CDs routinely requested at the NAC for patients suspected of having immunodeficiency. Patients were then enrolled in this audit if they met the diagnostic criteria for CPA previously described: (1) chronic (duration >3 months) pulmonary or systemic symptoms (e.g., productive cough, haemoptysis, dyspnoea, fatigue, weight loss), (2) radiological evidence of a progressive (over months or years) pulmonary lesion with surrounding inflammation (e.g., cavitation, infiltration, and pleural thickening), (3) no major apparent immunocompromising factors (e.g., AIDS, leukaemia, or transplantation), (4) serological or microbiological evidence of *Aspergillus*, and (5) absence of alternative diagnosis explaining the findings [26,27].

**Figure 1:**
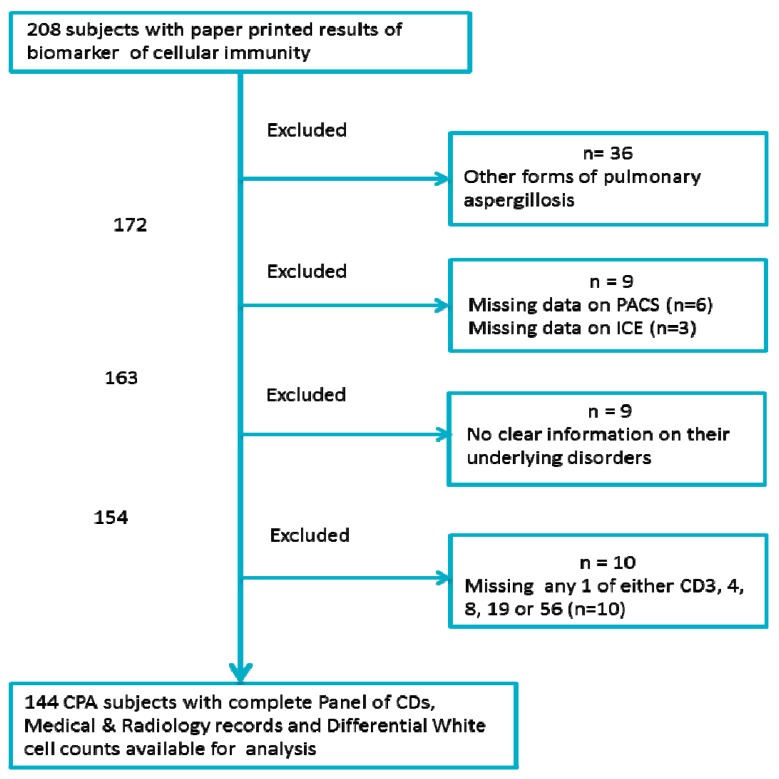
A flow chart showing the selection criteria for the 144 patients. ICE: Integrated Clinical Environment, PACS: Picture archiving and communication system, CD: Cluster of differentiation.

Only CPA patients with a complete panel of CD markers are included in the study. The other mandatory data required for inclusion were: (1) blood indices (total white cell, lymphocytes, neutrophils, and monocyte counts), (2) radiological information (computed tomography (CT) images and thoracic X-ray), (3) pertinent medical records indicating underlying pulmonary and systemic conditions, and (4) use of immunosuppressant medications. Only data available in written and electronic medical records was collected. Figure 1 shows the study schematic demonstrating the inclusion and exclusion criteria.

### 2.3. Immunology Data

CD3 (reference range (700–2100) × 10^9^/L), CD4 (reference range (300–1400) × 10^9^/L), CD8 (reference range (200–900) × 10^9^/L), CD19 (reference range (100–900) × 10^9^/L), and CD56 (reference range (90–600) × 10^9^/L) markers were done by the Department of Immunology, Central Manchester University Hospitals NHS Foundation Trust (CMFT) (Clinical Science Building 3, Manchester Royal Infirmary, Oxford Road, Manchester, UK). This facility is accredited (IS0 15189:2012) for CD enumeration using FC500 MPL flow cytometer equipment (Beckman Coulter Ltd., Buckinghamshire, UK). The dates of sampling were recorded. Absolute T- and B-lymphocytes and NK-cell CD counts were recorded; the laboratory does not provide percentage CDs, the reference ranges given are derived from healthy Europeans. Changes in reference ranges from 16th November 2015 were noted. The current reference ranges were used for statistical analyses, even for results produced before 16 November 2015. The unit of all CD biomarkers used in this study is (×10^9^/L).

### 2.4. Medical Records

We performed thorough hand searching of clinical case notes and correspondences. A standardised data collection sheet was used. Details recorded included confirmation of CPA diagnosis, age, and gender; date of enrolment into clinical care at NAC, underlying pulmonary disorder(s), if any, known systemic co-morbidities, chronic medication, especially immunosuppressive therapy, and band categorization.

### 2.5. Radiology

Electronic Images and reports were obtained from picture archiving and communication system (PACS) software accessed through the Centricity Enterprise Web version 3.0 (General Electric Healthcare, Barrington, IL, USA) at UHSM. Lung windows of both conventional and high resolution multi-slice thoracic computed tomography (CT) images with or without intravenous contrast administered, and plain chest X-ray images were electronically obtained. Baseline images at the time of diagnosis were examined. We also reviewed all the electronic reports of the images submitted by consultant radiologists. Data obtained included: (1) pulmonary involvement (unilateral or bilateral disease), (2) lobe(s) involved, (3) cavitation, (4) evidence of pleural thickening, and (5) presence of aspergilloma.

### 2.6. Complete Blood Count

Electronic diagnostic results were accessed through the integrated clinical environment (ICE) SunquestICE^®^ software package version 5.4.0 (Sunquest Information Systems (Europe), Ltd., Uxbridge, UK). Complete blood count (CBC) results were requested on the same day or 2 weeks before/after CD enumeration were recorded. Data collected included total white cell (reference range (4.0–11.0) × 10^3^/µL), neutrophils (reference range (2.0–7.5) × 10^3^/µL), lymphocytes (reference range (1.5–4.0) × 10^3^/µL), and monocyte (reference range (0.20–0.80) × 10^3^/µL) counts. For results more than 2 weeks before or after the CD enumeration, two different values were attained and their mean values were obtained. All CBCs were done by the clinical haematology department, UHSM. The unit of all CBC parameters used in this study is (×10^3^/µL).

### 2.7. Data Management

Data from the patients’ files and immunology reports were summarised and entered in a printed data collection sheet. The electronically retrieved data from SunquestICE^®^ desktop and PACS web version 3.0 software together with data on the data collection sheet were thereafter entered into Microsoft Excel 2010 spread sheet (Microsoft Corp., Redmond, WA, USA). The Excel workbook was password protected to restrict access to patients’ identifiable information or unlikely alteration of the collected data. Patients’ identifiable information entered into the Excel work book included the NHS and RM2 numbers, date of birth, and initials of their names. All this identifiable information was deleted after assigning study numbers to the subjects and prior to exportation to SPSS for statistical analysis. Encrypted flash drives were used for data transfer between the NHS trust computers, e-mailing of patients’ data was considered unacceptable.

### 2.8. Statistical Analysis

All statistical analyses were performed using statistical package for the social science (SPSS) software version 22.0 (IBM Corp., Armonk, NY, USA). Unless stated otherwise, statistical significance was at the 5% level for all analyses. Summary statistics were presented in terms of means, ranges, and standard deviations for continuous data. Non-normally distributed variables were presented in terms of medians and ranges. Pearson’s correlation was used to test the strength of association for normally distributed variables (CD3, CD4, and lymphocytes) and Spearman’s rank correlation was used if one or both variables were not normally distributed (CD8, CD19, and CD56). CD and CBC variables were dichotomised into low (CD19, CD56, CD4, CD8, CD4:CD8 ratio, lymphocyte counts) or high (total white cell count, neutrophils, CD4:CD8 ratio, monocytes) and normal. Pearson’s chi-squared tests and Fisher’s exact probability tests were performed as appropriate to assess for associations between the CDs, CBC parameters, and radiological and clinical (co-morbidities) characteristics. Logistic regression analyses were performed with multiple covariates to determine independent predictors. Odds ratios and 95% confidence intervals (CIs) were recorded.

### 2.9. Ethical Consideration

This audit was a service evaluation and, as such, informed consent was not required. However, all the Caldicott principles of transfer and handling of patients’ identifiable information were observed. The study was registered with the audit department at UHSM.

## 3. Results

### 3.1. Demographics, Baseline Radiological and Clinical Characteristics

One hundred and forty-four patients with complete data were analysed. Eighty-five patients were male (59%), and the median age at time of testing was 60 years (range: 22–84). Eighty-seven (60%) had unilateral disease with 64% (*n* = 56) of the unilateral disease affecting the right lungs of the patients. Ninety-one (63%) patients had multiple cavities in their lungs with fungal ball (aspergilloma) occupying cavities of up to 71 (49%) patients. Upper lobes were the most affected lobes in this cohort of patients (81%, *n* = 117).

The median number of the underlying conditions among these patients was 2 (range: 0–6). Two (1.4%) patients had no identifiable lung pathology. Previous mycobacterial (either classic or atypical) infection was the most frequent identified predisposing factor; at least 48 (33.3%) patients had documented previous TB (*n* = 31, 22%) and NTM (*n* = 17, 12%) infections. Bronchiectasis (*n* = 25, 17%), asthma (*n* = 24, 17%), COPD ± emphysema (*n* = 38, 26%), ABPA (*n* = 17, 12%), and previous lung surgeries (*n* = 17, 12) were also commonly identified. Other less common underlying conditions included pulmonary sarcoidosis (13, 9%), gamma interferon deficiency (12, 8%), previous pneumothorax (13, 9%), mannose binding lectin deficiency (10, 7%), survivors of lung cancer (7, 5%), rheumatoid arthritis (4, 3%), and impaired 1L-17 production (2, 1%). Underlying disorders such as community acquired pneumonia, connective tissue disorders, hypertension, diabetes, and other systemic disorders that were recorded constituted the remaining 44 risk factors of the total 274 underlying disorders. Patients’ demographic, radiological, and clinical characteristics are shown in Table 1 and Table 2 below.

### 3.2. Expression of CDs, Total and Differential White Cell Counts

The distribution of the CDs (CD3, CD4, CD8, CD19, and CD56), total white cell, neutrophil, lymphocyte, and monocyte counts in this group of patients are summarised in Table 3.

#### 3.2.1. CD4:CD8 Ratio

The laboratory reference range for CD4:CD8 ratio is 0.9–1.9 (Beckman Coulter (UK) Ltd.). The calculated mean CD4:CD8 ratio was 1.55 from our reference laboratory values of CD4 and CD8. A median CD4:CD8 ratio of 1.9 (range: 0.4–6.9) was obtained from the 144 patients. This was just at the upper limit of the reference range and well above the calculated mean CD4:CD8 ratio. Sixty-one (42%) patients had CD4:CD8 ratio within the reference range, 68 (47%) patients had ratios above 1.9, and only 15 (10%) patients had ratios of less than 0.9 (Table 3).

#### 3.2.2. Combined and Isolated Lymphopenia

**Figure 2:**
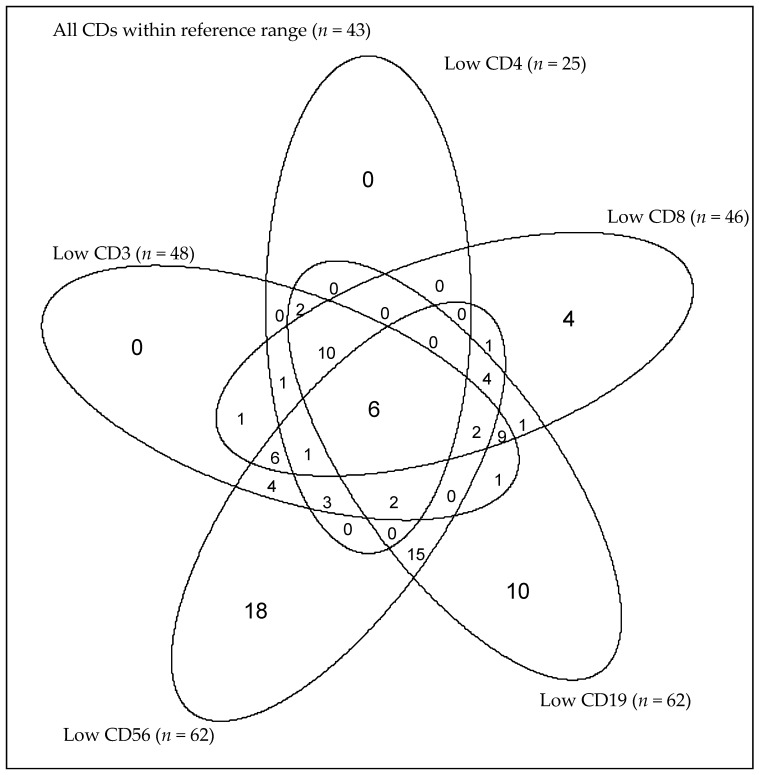
A Venn-diagram showing the distribution of all of the five CDs among the 144 patients. Six (4%) patients had lymphopenia in all the five CDs. Eighteen (13%), 10 (7%), and 15 (10%) patients had isolated CD56, CD19, and both CD56 and CD19 lymphopenia, respectively. Four (3%) patients had isolated CD8 lymphopenia. No patient had an isolated low CD4 or low CD3 count.

Six (4%) patients had combined CD3, CD4, CD8, CD19, and CD56 lymphopenia. Eighteen (13%), 10 (7%), and 15 (10%) of the 144 CPA patients had isolated CD56, CD19, and both CD56 and CD19 lymphopenia, respectively (Table 4). The mean monocyte and lymphocyte counts for this group of patients are within or near the lower limit of the reference ranges. Only four (3%) patients had isolated CD8 lymphopenia (Table 4). No patient had an isolated low CD4 or low CD3 count; all were associated with subnormal CD8 or CD19 (Figure 2).

#### 3.2.3. Correlation between CDs and CBC Parameters

Unsurprisingly, there was a strong significant positive correlation between CD3 and CD4 counts (Pearson’s coefficient, *r*^2^ = 0.91, *p* < 0.001), CD3 and CD8 (Spearman’s rho (ρ) = 0.84, *p* < 0.001), and CD3 and lymphocyte count (*r*^2^ = 0.77, *p* < 0.001). There was also a marked association between CD4 and CD8 (ρ = 0.67, *p* < 0.001), CD19 and lymphocytes (ρ = 0.54, *p* < 0.001), CD4 and CD19 (ρ = 0.52, *p* < 0.001), CD3 and CD19 (ρ = 0.49, *p* < 0.001), and CD8 and CD19 (ρ = 0.42, *p* < 0.001).

However, there was no statistically significant association between CD3 and CD56 (ρ = 0.10, *p* = 0.21), CD19 and CD56 (ρ = 0.15, *p* = 0.077), monocytes and CD56 (ρ = 0.16, *p* = 0.055), or neutrophils and CD56 (ρ = 0.13, *p* = 0.12).

### 3.3. Association between CDs and CBC Parameters, Underlying Disorders, Demographic and Radiological Characteristics

Pulmonary sarcoidosis was associated with a low CD19 count: 10 (77%) versus 52 (40%) without (*p* = 0.01). Likewise, emphysema was also associated with a low CD19 count: 11 (73%) versus 51 (40%) without emphysema (*p* = 0.012). On multivariable logistic analysis, statistically significant independent associations were observed between low CD19 and pulmonary sarcoidosis (odds ratio, 5.53; 95% CI, 1.43–21.33; *p* = 0.013), and emphysema (odds ratio, 4.58; 95% CI, 1.36–15.38; *p* = 0.014).

A low CD56 count appears protective for bronchiectasis: 5 (20%) versus 57 (48%) of patients without bronchiectasis (*p* = 0.010). A low CD56 count was seen more often in patients with a previous pneumothorax: 9 (69%) versus 53 (41%) patients with no prior pneumothorax (*p* = 0.046). Multivariable logistics analysis showed a protective role of low CD56 in bronchiectasis (OR, 0.27; 95% CI, 0.10–0.77; *p* = 0.014).

Pulmonary sarcoidosis was associated with a low CD3 count: 9 (69%) versus 39 (30%) without (Fisher’s exact test *p =* 0.010). A low CD3 count was also associated with previously treated lung cancer: 5 (71%) of the lung cancer survivors compared to 43 (31%) of the patients who were not previously treated for lung cancer (Fisher’s exact test *p =* 0.041). Lastly, multicavitary disease: 38 (42%) versus 10 (19%) in patients with single cavities or nodules (*p =* 0.005). Multicavitary CPA disease (OR, 2.95; 95% CI, 1.30–6.72; *p =* 0.010) and pulmonary sarcoidosis (OR, 4.94; 95% CI, 1.39–17.57; *p =* 0.014) were significantly associated with low CD3.

A low CD4 count was more often seen in patients with rheumatoid arthritis: 3 (75%) compared to 22 (16%) of patients without rheumatoid arthritis (Fisher’s exact test *p =* 0.017). Likewise, IL-17 deficiency (2 (100%) compared to 23 (16%) of patients with normal IL-17 cytokine levels) was associated with a low CD4 count (Fisher’s exact test *p =* 0.029). Pulmonary sarcoidosis was also associated with a low CD4 count: 8 (62%) patients versus 17 (13.0%) of the patients without pulmonary sarcoidosis (Fisher’s exact test *p* < 0.001). Multivariable logistic analysis did not reveal any statistically significant associations between low CD4 and the above factors.

Pulmonary sarcoidosis was associated with lymphopenia, where 11 (85%) of the 13 patients had low lymphocyte counts compared to 73 (56%) without pulmonary sarcoidosis (*p =* 0.044). Age was negatively associated with CD3, CD8 and lymphocyte counts. However, these associations were not statistically significant (*p =* 0.244, *p =* 0.703, *p =* 0.401, respectively) There were no significant associations elicited between the CDs and gender, presence or absence of aspergilloma, pleural thickening, or lung involvement.

## 4. Discussion

In this study, we established that significant CD3, CD4, CD8, CD19, and CD56 lymphopenia occurs in patients with CPA. Fifteen (10%) patients without documented HIV infection had very severe CD4 suppression, that is, CD4 <200 cells/µL. Furthermore, we found poor immune homeostasis with immune response inclined towards inflammatory signals, as shown by high CD4:CD8 ratio among these patients. Our findings are in keeping with the currently budding concept that mild immunological suppression and genetic polymorphisms are important factors leading to the development of CPA [12,23]. Severe immunosuppression in the form of chronic granulomatous disease (CGD) and profound neutropenia is well known to predispose to invasive pulmonary aspergillosis [28] and so is T_H_2 biased immune mediated pathological inflammation in ABPA [29].

To our knowledge, we present for the first time in literature a study that describes the expression of biomarkers of T-cells (CD3, CD4, and CD8), B-cells (CD19), and natural killer cells (CD56) in a large population of patients with CPA.

It is striking to note that the majority of patients had just one or two identifiable underlying diseases and two (1.4%) patients had no identifiable risk factors. In these patients, one might expect non-pulmonary systemic factors, possibly genetic or immune defects, to play a role in the development of CPA. Interestingly, patients previously treated for cancer (cancer survivors) had lower CD3 counts than other patients, suggesting a prolonged immune suppressing effect following cancer treatment (or a pre-disposition to lung cancer) that could contribute to CPA development. In addition, a low CD3 count was associated with multicavitary as opposed to single cavitary disease. Therefore, more immunosuppression may lead to more extensive disease.

A substantial number of patients had subnormal counts of individual CD subsets. Table 4 shows the numbers of such patients, which was most frequently seen for CD56 (13%), followed by CD19 (7%). In both cases, the total lymphocyte count was usually in the normal range, although not always. Combined low CD19 and CD56 counts were seen in an additional 15 patients (10%), also with generally normal lymphocyte counts, and therefore subnormal CD19 and/or CD56 counts were found in 43 patients (30%). Isolated subnormal CD8 counts were seen in only four patients (3%), and none had isolated subnormal CD4 or CD3. While “compensatory” monocytosis was seen in some patients, this was by no means universal, and no patients had monocytopenia. No published literature currently exists on these cellular biomarkers in CPA patients to compare with our results. Our findings suggest multiple heterogeneity and likely several pathways to CPA development.

CD3, CD4, CD8, and CD19 lymphocyte subset counts were significantly correlated (ƿ ranged from 0.42 to 0.92, all *p* < 0.001), indicating severe overall T-cell and B-cell lymphopenia in the peripheral blood of these patients and inadequate cellular and humoral responses in the adaptive arm of the immune system. However, there was no statistically significant correlation between CD3 and CD56 counts (ƿ = 0.10, *p =* 0.21) and CD19 and CD56 counts (ƿ = 0.15, *p =* 0.077). This is theoretically expected, as CD56 and the other CDs in this study are from different lineages. However, more significantly, it provides an insight that deficits in both innate and adaptive arms of the immune system are involved in the pathogenesis of CPA.

In our study, 13 (9%) patients had a histologically proven diagnosis of pulmonary sarcoidosis, comparable to 7.1% in a previous study at the same centre [11]. Ten (77%) of those with sarcoidosis in our study had low CD19 counts, nine (69%) had low CD3 counts, and eight (62%) had low CD4 counts. Sweiss et al. (2010) reported significant lymphopenia involving CD4, CD8, and CD19 positive cells among sarcoidosis patients, and this was unrelated to their medical treatment but rather more to disease pathology [30]. CPA complicates sarcoidosis, with estimate global burden of over 70,000 individuals [31]. However, no statistically significant CD56, CD8 lymphopenia was seen among CPA patients with sarcoidosis in our study, in contrast to the work of Sweiss and colleagues.

In our study, impaired IL-17 production was statistically associated with low CD4 (Fisher’s exact test *p =* 0.029). This is consistent with the experimental work of Doffinger et al. (2014) that showed a significant impairment of IFN-γ, IL-12, and IL-17 production in a majority of patients with CPA, suggesting a major involvement of T_H_1/T_H_17 and potentially NK-cell subsets [32]. IL-12, also known as T-cell stimulating factor, is produced by antigen presenting cells, in particular dendritic cells and macrophages, and neutrophils to a lesser extent. It stimulates the differentiation and production of IFN-γ and TNF-α by naive T-cells and natural killer cells [33]. Both CD4^+^ T_H_1 cells and CD8^+^ T-cells produce IFN-γ, which is critical in host defence against pulmonary infections [17].

Prior TB and NTM infection were not statistically associated with CD4 or CD8 lymphopenia. Active TB infection is known to cause CD4 and CD8 lymphopenia, and T-cell exhaustion and depletion [18,34]. Loss of CD4^+^ T-cells results in progressive primary TB infection, reactivation of latent TB infection (LTBI), and enhanced susceptibility to re-infection but not susceptibly to TB [34]. Both CD4^+^ and CD8^+^ T-cells have been reported to recover towards normality after successful anti-TB treatment [35]. T-cell exhaustion is a known syndrome implicated in a number of chronic infections [36]. T-cell exhaustion has not been previously reported in CPA. Perhaps the profound lymphopenia indicates a possibility of such a phenomenon in CPA. Recently, clinical studies have shown that T-cell immunity impairment is associated with an increased susceptibility to *A. fumigatus* infection [16].

Sixty-eight (47%) patients had high CD4:CD8 ratio, an indication of a pro-inflammatory biased immune signal among these patients. CD4^+^ T-lymphocytes are important mediators of host inflammatory/anti-inflammatory responses, with the balance between T_H_1/T_H_17 and T_H_2 phenotypes dictating disease pathology [37]. *Aspergillus*-host interactions trigger the cells of the innate immune system that subsequently elicit the adaptive arm to regulate the balance between pro-inflammatory and anti-inflammatory signals [14]. Thirty-eight (26.4%) of our patients had neutrophilia. Neutrophils and alveolar macrophages are important human innate cells against *Aspergillus* infection through phagocytosis and secretion of the neutrophil extracellular traps (NETs) [38]. An experimental study by Smith et al. (2015) showed increased levels of macrophage-derived neutrophil chemoattractant pro-platelet basic proteins, which is thought to be pathological in ABPA and CCPA patients [39].

Sixty-two (43%) patients had low CD19 counts, demonstrating profound humoral immune deficiency. Contrarily, over 90% of CPA patients have positive *Aspergillus*-specific IgG, a key criterion for diagnosis [26,40,41]. The possible explanation here could be that the few patients with negative anti-*Aspergillus*-specific IgG antibody tests had very low CD19 counts. We used absolute CD19 values below reference range as “low”, but this might not be critically low enough to impair antibody production. Page et al. (2016) reported two cases without any *Aspergillus* IgG in multiple assays and referred to them as “seronegative CPA”, since they had overt CPA but negative titres for *Aspergillus* antibody; ineffective antibody response to *Aspergillus* due to underlying immune deficit was a suggested explanation [42]. In a recent study, only 47.6% of our CPA patients were shown to have adequate response 3 months after administration of pneumococcal 23-valent polysaccharide vaccine [43].

Severe asthma and allergic bronchopulmonary aspergillosis (ABPA) are risk factors for development of CPA in a minority of our patients [11]. Consistent with our findings, a recent study has shown no significant difference in CD3, CD8, and CD56 expression in patients with asthma and healthy controls [44].

This study, however, has a number of limitations. First, absolute T- and B-lymphocyte counts can be influenced by many factors such as infections and medications compared to the relatively stable percentage total lymphocyte counts [45]. CPA is a chronic infection; therefore, it cannot be established if CPA is the cause or the consequence of the immunological alterations. Second, with the exception of CD4 counts, it is difficult to establish precise levels of clinically significant CD3, CD8, CD19, and CD56 lymphopenia. This makes it difficult to translate these results in clinical practice, as clinically relevant depression in CD counts might be quite different from the lower limit of the laboratory reference range. Third, the retrospective nature of the study made it difficult to access other potentially vital information such as concomitant corticosteroid or immunosuppressive medications (although infrequently used in our patients). However, significant immunosuppression is an exclusion from the diagnosis of CPA, so even if present, such medications are unlikely to have a radical impact on the results. Fourth, some immunological abnormalities are associated with underlying pulmonary disorders, including sarcoidosis, but these parameters have not been well studied in other underlying disorders for CPA. Therefore, the specificity of the findings requires substantial additional work. Fifth, only a single biomarker of natural killer cells (CD56) and B-cells (CD19) were used in this study and yet CD16 and CD57 are also expressed by NK-cells and CD20 and CD22 for B-cells. Lastly, we cannot exclude all systemic co-morbidities if not already known by the referring physician.

The present study is the first of its kind to provide direct evidence of considerable defects in both the innate and adaptive arm of the immune system in the majority of patients with CPA. Routine analyses of these biomarkers, preferably both absolute and percentage T- and B-lymphocyte counts are advised to detect subtle immunological deficits in this group of patients. This could help tailor individualised management of CPA patients.

This study could benefit in the future design of a matched prospective study to evaluate the trend, prognostic and therapeutic significance of these biomarkers in patients with CPA compared to patients with underlying pulmonary conditions that do not have CPA. Particular patient groups within CPA need further study, notably those who are seronegative for *Aspergillus* IgG.

## Figures and Tables

**Table 1:** Demographic and radiographic characteristics.

Characteristic	Number	Percent
**Gender**		
Male	85	59.0
Female	59	41.0
**Pleural thickening**		
Yes	91	63.2
No	53	36.8
**Cavitation**		
Multicavitary	91	63.2
Single cavity or *Aspergillus* nodule	53	36.8
**Aspergilloma**		
Yes	71	49.3
No	73	50.7
**Lung involvement**		
Bilateral	57	39.6
Unilateral	87	60.4
Left Unilateral	31	21.5
Right Unilateral	56	38.9
**Lobes involved**		
Upper lobes	117	81.3
Middle lobe	11	7.6
Lower lobe	16	11.1

**Table 2:** Underlying conditions among the 144 patients with chronic pulmonary aspergillosis.

Underlying Disorder	Number	Percent
Tuberculosis	31	21.5
Bronchiectasis	25	17.4
Asthma	24	16.7
Chronic obstructive pulmonary diseases	23	16.0
Non-tuberculous mycobacterial infection	17	11.8
Allergic bronchopulmonary aspergillosis	17	11.8
Previous lung surgery	17	11.8
Emphysema	15	10.4
Sarcoidosis	13	9.0
Pneumothorax	13	9.0
Gamma interferon deficiency	12	8.3
Mannose binding lectin-deficiency	10	6.9
Lung cancer survivors	7	4.9
Rheumatoid arthritis	4	2.8
Impaired interleukin-17 production	2	1.4
**Total**	**230**	**159.7%**

**Table 3:** Summary of results of CDs, total and differential white cell count parameters.

Parameter (Reference Range *)	Mean or Median	Range	Standard Deviation	Below the Reference Range *n* (%)	Within the Reference Range *n* (%)	Above Reference Range *n* (%)
CD3 (700–2100)	1019	73–2684	563	48 (33.3)	90 (62.5)	6 (4.2)
CD4 (300–1400)	652	36–1815	361	25 (17.4)	112 (77.8)	7 (4.9)
CD4 <200	-	-	-	15 (10.4)	-	-
CD8 (200–900)	312	25–1415	-	46 (31.9)	89 (61.8)	9 (6.3)
CD19 (100–500)	114	11–1234	-	62 (43.1)	79 (54.2)	4 (2.8)
CD56 (90–600)	113	2–556	-	62 (43.1)	82 (56.9)	0 (0.0)
CD4:CD8 Ratio (0.9–1.9)	2.1	0.4–6.9	1.2	15 (10.4)	61 (42.4)	68 (47.2)
White cell count (4–11)	8.2	3.9–22.2	-	1 (0.7)	115 (79.9)	28 (19.4)
Neutrophils (2.0–7.5)	5.4	2.1–19.8	-	0 (0.0)	106 (73.6)	38 (26.4)
Lymphocytes (1.5–4.0)	1.5	0.2–3.2	0.30	84 (58.3)	60 (41.7)	0 (0.0)
Monocytes (0.2–0.8)	0.62	0.13–1.67	-	2 (1.4)	99 (68.8)	43 (29.9)

* Reference based on healthy European volunteers.

**Table 4:** Patients with isolated subnormal levels of CD56, CD19, CD8, and CD4, as well as both CD19 and CD56, with corresponding lymphocyte and monocyte counts in those patients.

Biomarker ^#^	*N* (%)	Mean CDs (Range)	Mean Lymphocytes (Range)	Mean Monocytes (Range)
CD56	18 (12.5)	61 (21–86)	2.0 (1.35–2.98)	0.66 (0.33–1.04)
CD19	10 (6.9)	76 (25–96)	1.63 (0.98–2.51)	0.82 (0.21–1.67)
CD56 and CD19	15 (10.4)	–	1.6 (1.01–2.73)	0.69 (0.43–1.35)
CD8	4 (2.8)	155 (111–178)	1.21 (1.12–1.27)	0.55 (0.39–0.71)
CD4	0	–	–	–
CD3	0	–	–	–

^#^ Reference ranges: CD3 (700–2100), CD4 (300–1400), CD8 (200–900), CD19 (100–500), CD56 (90–600), Monocytes: 0.2–0.8: Lymphocytes: 1.5–4.

## References

[1] Kwon-Chung K.J., Sugui J.A. (2013). *Aspergillus fumigatus*-What Makes the Species a Ubiquitous Human Fungal Pathogen?. PLoS Pathog..

[2] Denning D.W. (1998). Invasive Aspergillosis. Clin. Infect. Dis..

[3] Hope W.W., Walsh T.J., Denning D.W. (2005). The invasive and saprophytic syndromes due to *Aspergillus* spp.. Med. Mycol..

[4] Segal B.H. (2009). Aspergillosis. N. Engl. J. Med..

[5] Agarwal R., Chakrabarti A., Shah A., Gupta D., Meis J.F., Guleria R., Moss R., Denning D.W., ABPA complicating asthma ISHAM working group (2013). Allergic bronchopulmonary aspergillosis: Review of literature and proposal of new diagnostic and classification criteria. Clin. Exp. Allergy.

[6] Denning D.W. (2001). Chronic forms of pulmonary aspergillosis. Clin. Microbiol. Infect..

[7] Kosmidis C., Denning D.W. (2015). The clinical spectrum of pulmonary aspergillosis. Thorax.

[8] Patterson K.C., Strek M.E. (2014). Diagnosis and treatment of pulmonary aspergillosis syndromes. Chest.

[9] Denning D.W., Cadranel J., Beigelman-Aubry C., Ader F., Chakrabarti A., Blot S., Ullmann A.J., Dimopoulos G., Lange C., European Society for Clinical Microbiology and Infectious Diseases and European Respiratory Society (2016). Chronic pulmonary aspergillosis: Rationale and clinical guidelines for diagnosis and management. Eur. Respir. J..

[10] Camuset J., Lavole A., Wislez M., Khalil A., Bellocq A., Bazelly B., Mayaud C., Cadranel J. (2007). Bronchopulmonary aspergillosis infections in the non-immunocompromised patient. Revue de Pneumologie Clinique.

[11] Smith N.L., Denning D.W. (2011). Underlying conditions in chronic pulmonary aspergillosis including simple aspergilloma. Eur. Respir. J..

[12] Hayes G.E., Novak-Frazer L. (2016). Chronic Pulmonary Aspergillosis—Where Are We? and Where Are We Going?. J. Fungi.

[13] Espinosa V., Rivera A. (2016). First line of defense: Innate cell-mediated control of pulmonary Aspergillosis. Front. Microbiol..

[14] Park S.J., Mehrad B. (2009). Innate immunity to Aspergillus species. Clin. Microbiol. Rev..

[15] Beitzen-Heineke A., Bouzani M., Schmitt A.L. (2016). Human Invariant Natural Killer T cells possess immune-modulating functions during Aspergillus infection. Medical.

[16] Camargo J.F., Husain S. (2014). Immune correlates of protection in human invasive aspergillosis. Clin. Infect. Dis..

[17] Romani L. (2011). Immunity to fungal infections. Nat. Rev. Immunol..

[18] Murphy K.P., Travers P., Walport M., Janeway C. (2008). Janeway’s Immuno Biology.

[19] Jolink H., De Boer R., Willems L.N.A., Van D.J.T., Falkenburg J.H.F., Heemskerk M.H.M. (2015). T helper 2 response in allergic bronchopulmonary aspergillosis is not driven by specific Aspergillus antigens. Allergy Eur. J. Allergy Clin. Immunol..

[20] Harrison E., Singh A., Morris J., Smith N.L., Fraczek M.G., Moore C.B., Denning D.W. (2012). Mannose-binding lectin genotype and serum levels in patients with chronic and allergic pulmonary aspergillosis. Int. J. Immunogenet..

[21] Koldehoff M., Beelen D.W., Elmaagacli A.H. (2013). Increased susceptibility for aspergillosis and post-transplant immune deficiency in patients with gene variants of TLR4 after stem cell transplantation. Transpl. Infect. Dis..

[22] Sainz J., Hassan L., Perez E., Romero A., Moratalla A., López-Fernández E., Oyonarte S., Jurado M. (2007). Interleukin-10 promoter polymorphism as risk factor to develop invasive pulmonary aspergillosis. Immunol. Lett..

[23] Smith N.L.D., Denning D.W. (2014). Clinical implications of interferon-γ genetic and epigenetic variants. Immunology.

[24] Kelleher P., Goodsall A., Mulgirigama A., Kunst H., Henderson D.C., Wilson R., Newman-Taylor A., Levin M. (2006). Interferon-γ therapy in two patients with progressive chronic pulmonary aspergillosis. Eur. Respir. J..

[25] Smith N.L.D., Hankinson J., Simpson A., Denning D.W., Bowyer P. (2014). Reduced expression of TLR3, TLR10 and TREM1 by human macrophages in Chronic cavitary pulmonary aspergillosis, and novel associations of VEGFA, DENND1B and PLAT. Clin. Microbiol. Infect..

[26] Denning D.W., Riniotis K., Dobrashian R., Sambatakou H. (2003). Chronic Cavitary and Fibrosing Pulmonary and Pleural Aspergillosis: Case Series, Proposed Nomenclature Change, and Review. Clin. Infect. Dis..

[27] Patterson T.F., Thompson G.R., Denning D.W., Fishman J.A., Hadley S., Herbrecht R., Kontoyiannis D.P., Marr K.A., Morrison V.A., Nguyen M.H. (2016). Practice Guidelines for the Diagnosis and Management of Aspergillosis: 2016 Update by the Infectious Diseases Society of America. Clin. Infect. Dis..

[28] Segal B.H., Romani L.R. (2009). Invasive aspergillosis in chronic granulomatous disease. Med. Mycol..

[29] Skov M., Poulsen L.K., Koch C. (1999). Increased antigen-specific Th-2 response in allergic bronchopulmonary aspergillosis (ABPA) in patients with cystic fibrosis. Pediatr. Pulmonol..

[30] Sweiss N.J., Salloum R., Ghandi S., Alegre M.L., Sawaqed R., Badaracco M., Pursell K., Pitrak D., Baughman R.P., Moller D.R. (2010). Significant CD4, CD8, and CD19 lymphopenia in peripheral blood of sarcoidosis patients correlates with severe disease manifestations. PLoS ONE.

[31] Denning D.W., Pleuvry A., Cole D.C. (2013). Global burden of chronic pulmonary aspergillosis complicating sarcoidosis. Eur. Respir. J..

[32] Döffinger R., Harris C., Lear S., Newton P., Alachkar H., Dinakantha S. (2014). Impaired Th1 and Th17 immunity in chronic pulmonary aspergillosis. Proceedings of the 6th Advances against Aspergillosis.

[33] Dorman S.E., Holland S.M. (2000). Interferon-γ and interleukin-12 pathway defects and human disease. Cytokine Growth Factor Rev..

[34] Jayaraman P., Jacques M.K., Zhu C., Steblenko K.M., Stowell B.L., Madi A., Anderson A.C., Kuchroo V.K., Behar S.M. (2016). TIM3 Mediates T Cell Exhaustion during Mycobacterium tuberculosis Infection. PLoS Pathog..

[35] Al-Aska A., Al-Anazi A.R., Al-Subaei S.S., Al-Hedaithy M.A., Barry M.A., Somily A.M., Buba F., Yusuf U., Al Anazi N.A. (2011). CD4+ T-lymphopenia in HIV negative tuberculous patients at King Khalid University Hospital in Riyadh, Saudi Arabia. Eur. J. Med. Res..

[36] Wherry E.J. (2011). T cell exhaustion. Nat. Immunol..

[37] Carvalho A., Cunha C., Iannitti R.G., De Luca A., Giovannini G., Bistoni F., Romani L. (2012). Inflammation in aspergillosis: The good, the bad, and the therapeutic. Ann. N. Y. Acad. Sci..

[38] Hasenberg M., Behnsen J., Krappmann S., Brakhage A., Gunzer M. (2011). Phagocyte responses towards Aspergillus fumigatus. Int. J. Med. Microbiol..

[39] Smith N.L.D., Bromley M.J., Denning D.W., Simpson A., Bowyer P. (2015). Elevated levels of the neutrophil chemoattractant pro-platelet basic protein in macrophages from individuals with chronic and allergic aspergillosis. J. Infect. Dis..

[40] Uffredi M.L., Mangiapan G., Cadranel J., Kac G. (2003). Significance of *Aspergillus fumigatus* isolation from respiratory specimens of nongranulocytopenic patients. Eur. J. Clin. Microbiol. Infect. Dis..

[41] Ohba H., Miwa S., Shirai M., Kanai M., Eifuku T., Suda T., Hayakawa H., Chida K. (2012). Clinical characteristics and prognosis of chronic pulmonary aspergillosis. Respir. Med..

[42] Page I.D., Richardson M.D., Denning D.W. (2016). Comparison of six Aspergillus-specific IgG assays for the diagnosis of chronic pulmonary aspergillosis (CPA). J. Infect..

[43] Kosmidis C., Powell G., Borrow R., Morris J., Alachkar H., Denning D.W. (2015). Response to pneumococcal polysaccharide vaccination in patients with chronic and allergic aspergillosis. Vaccine.

[44] Tubby C., Negm O.H., Harrison T., Tighe P.J., Todd I., Fairclough L.C. (2016). Peripheral killer cells do not differentiate between asthma patients with or without fixed airway obstruction. J. Asthma.

[45] Slade J.D., Hepburn B. (1983). Prednisone-induced alterations of circulating human lymphocyte subsets. J. Lab. Clin. Meid..

